# Myofascial System and Physical Exercise: A Narrative Review on Stretching (Part I)

**DOI:** 10.7759/cureus.75077

**Published:** 2024-12-04

**Authors:** Saverio Colonna, Fabio Casacci

**Affiliations:** 1 Rehabilitation Medicine, Spine Center, Bologna, ITA; 2 Research and Development, Osteopathic Spine Center Education, Bologna, ITA

**Keywords:** fascial system, hill’s three-element model, muscle stretching exercise, myofascial pain, myofascial trigger points, proprioceptive neuromuscular facilitation (pnf), therapeutic fascial stretching

## Abstract

Over the past 20-30 years, numerous studies have expanded our understanding of the connective components within the human musculoskeletal system. The term "fascia" and, more specifically, the "fascial system" encompass a variety of connective tissues that perform multiple functions. Given the extensive scope of the topic of fascia and the fascial system, which cannot be fully addressed in a single article, this work will focus specifically on the role of fascia in tension transmission (mechanotransduction). This includes both the tensions generated by the contractile muscular component and the elastic recoil, which contribute to movement and posture in the human body.

A functional alteration of the fascia, due to the high density of receptors within it, can trigger pain symptoms identified as myofascial pain; this typically manifests at so-called myofascial trigger points (MTrPs). This article presents a new hypothesis on how MTrPs may develop as a result of imbalanced tension loading on the fascial components arranged in series and parallel to the contractile muscular component. While the literature contains several studies on the manual manipulation of fascia, limited evidence is available regarding the treatment of fascial dysfunctions through alternative modalities, such as therapeutic exercises aimed at lengthening or shortening these structures.

This work is divided into two parts: the first section provides an overview of the composition of fascia used for the transmission of tension forces and introduces the basis of the approach, using stretching techniques, to address dysfunctions related to excessive rigidity in the myofascial system. Additionally, a mechanical physiological basis is proposed for the various stretching methods described in the literature. The second part addresses the treatment of dysfunctions related to reduced rigidity in the fascial system through therapeutic exercise. The fascial training recommendations provided in this article are aimed at preventing and treating musculoskeletal disorders. They should be integrated with muscle-strengthening work, cardiovascular training, and coordination exercises.

Developing a fascial network in the body that is both flexible and resistant to injury involves applying recent discoveries from the rapidly advancing field of fascia research into effective training programs. This paper aims to motivate physiotherapists, osteopaths, sports trainers, and other movement instructors to integrate these principles and adapt them within their professional practices.

## Introduction and background

Until recently, it was common practice to categorize musculoskeletal disorders as a single entity [1]. As our knowledge of the human body has improved, etiopathogenetic differences between disorders affecting various components of the musculoskeletal structure have emerged [2]. Researchers used to focus on the physiology and pathology of certain muscle components and the therapeutic interventions targeting them [3]. However, research results from the past 20-30 years [2] suggest it is a mistake to treat contractile muscle tissue as a standalone entity separate from connective tissue, even if the relationship is not yet clear [2]. Recent findings indicate that what has traditionally been identified as muscle is, in fact, largely composed of connective tissue, which we now refer to as fascia, and which plays a fundamental role [4].

## Review

An introduction to fascia

As stated in a recent article [5], an examination of the literature available on PubMed shows that the earliest mention of fascia in the medical and clinical field dates back to 1814 [6], while the term "fasciæ" appears in a journal from 1824 [7]. According to the American Heritage Stedman’s Medical Dictionary [8], fascia is defined as "a sheet or band of fibrous connective tissue enveloping, separating, or binding together muscles, organs, and other soft structures of the body." Therefore, only well-defined fibrous connective tissue layers should properly be referred to as fascia, and it is inaccurate to use this term to describe all connective tissues in the body.

Fascia is a viscoelastic tissue that forms an uninterrupted, three-dimensional collagen matrix [9]. This tissue permeates the entire body, surrounding, supporting, protecting, connecting, and dividing the multiple muscular and visceral components of the organism [10]. Fascia performs several physiological and functional roles related to joint stability, general movement coordination, proprioception, and nociception [11]. Most importantly, it is responsible for transmitting mechanical forces (mechanotransduction) [12-14]. Due to its presence in every tissue at multiple hierarchical levels, fascia represents the main structural interconnective element, both externally and internally, between the different constituents of the human body. Huijing et al. [15] found that only 70% of muscle tension is transferred through tendons, which confirms their mechanical role. However, the remaining 30% of muscle force is conveyed to the connective tissue surrounding muscles, underscoring the deep fasciae's function in coordinating agonist, antagonist, and synergistic muscles at the peripheral level.

Fascia and fascia system classification

In the literature, various classifications of fascia exist [10]. For example, in an attempt to organize the nomenclature for fascial structures provided by the Federative International Committee on Anatomical Terminology (FICAT) [16], a functional classification system was developed that includes four categories of fascia: connecting, fascicular, compressive, and separating fascia. Each category was developed from descriptions in the literature on macroscopic anatomy, histology, and biomechanics; the category names reflect the function of the fascia. More recently, the Foundation of Osteopathic Research and Clinical Endorsement (FORCE), an organization that brings together various scientific figures from a multidisciplinary perspective, tried to find a common nomenclature that can be shared, starting from the scientific notions currently available [5]. Additionally, the Fascia Nomenclature Committee has proposed the following definition for the term fascia: "A fascia is a sheath, a sheet, or any other dissectible aggregations of connective tissue that forms beneath the skin to attach, enclose, and separate muscles and other internal organs” [17]. Instead, the final definition of the Fascial System proposed by a subcommittee of five members from the Fascia Nomenclature Committee is: "The fascial system consists of the three-dimensional continuum of soft, collagen-containing, loose and dense fibrous connective tissues that permeate the body. It incorporates elements such as adipose tissue, adventitiae and neurovascular sheaths, aponeuroses, deep and superficial fasciae, epineurium, joint capsules, ligaments, membranes, meninges, myofascial expansions, periostea, retinacula, septa, tendons, visceral fasciae, and all the intramuscular and intermuscular connective tissues including endo-/peri-/epimysium” [17]. 

For the purpose of this work, we will consider the simplified subdivision by Stecco et al. [18], an Italian researcher who has significantly contributed to the study of fascial systems. He classifies the simple fascial component involved in mechanotransduction into three main areas: (a) Superficial: This fascial layer is closest to the body’s surface and includes tissues such as the superficial fascia of the body and the subcutaneous fascia. (b) Deep: This fascial layer is located deeper than the first layer and includes structures such as the deep muscle fascia and the epimysial fascia that envelop the muscles. (c) Visceral: This is the innermost of the three fascial layers and surrounds the internal organs, providing structural support and facilitating organ movements within the body. The deep fascia is a continuous layer running from the trunk through to the upper and lower limbs and is considered the key element for transmitting loads in parallel, bypassing the joints [19].

The focus of this work will primarily be on the fascia involved in the transmission of tension essential for body movement, such as that produced by muscles, as it provides the insertion and/or origin for all muscle fibers, with an estimated percentage of approximately 30% [19].

Composition of fascia

Fascia is an uninterrupted structure composed of layers of dense connective tissue (type I and III collagen) interfaced with loose connective tissue. It exhibits the typical properties of viscoelastic structures [3]. In addition to fibers and cells, connective tissue systems also comprise an extracellular matrix (ECM) [20]. This ECM surrounds cells, protects them, holds them together, and also provides physical and biochemical signals that play a key regulatory role in determining the shape and activities of a cell. It can take various forms in different tissues but is generally composed of similar fibrous (non-globular) macromolecules secreted into the extracellular space, where they self-assemble. The macromolecules that make up the ECM are mainly glycoproteins; in animals, collagen is by far the most abundant protein. In addition to collagen, the matrix also contains proteoglycans, fibronectin, and laminin. According to Bordoni et al. [21], it is important to include the liquid component (liquid fascia) as part of the fascia's composition.

Myofascial pain

The "motor unit" or "muscle" has traditionally been seen as the fundamental unit in movement control. However, recent research [18] has highlighted the significant interaction between muscle fibers and intramuscular connective tissue, as well as between muscles and fasciae, indicating that muscles alone cannot be regarded as the sole organizers of movement. Additionally, muscle innervation and blood supply are closely linked with intramuscular connective tissue [22]. This realization led Luigi Stecco, in 2002 [22], to coin the term "myofascial unit" to emphasize the interconnected relationship, both anatomically and functionally, between fascia, muscles, and their associated structures.

The stiffening of connective tissue is a feature of various painful syndromes [23]. Indeed, localized increases in fascial rigidity are observed in certain "muscular" contractures - an abnormal increase in the passive stiffness of muscles - which are marked by dense connective tissue abundant in myofibroblasts and often linked to ongoing inflammatory processes [23]. Some studies have clarified the mechanisms by which stretching, applied to conditions of myofascial stiffening, can improve the range of motion (ROM) and reduce pain, as reported by a review study [24]. The painful syndromes most frequently associated with the stiffening of localized connective tissue include compartment syndrome of the lower leg induced by exercise, runner's knee (iliotibial friction band), tennis/golfer's elbow, frozen shoulder, plantar fasciitis, and Dupuytren's disease [23]. Other examples of conditions caused by localized stiffness that can manifest even without pain include scoliosis and high-arched feet. General conditions of increased stiffness include spastic paralysis such as after a stroke, neuromuscular diseases such as Parkinson's disease, and autoimmune diseases such as rheumatoid arthritis or scleroderma.

In these pathological conditions, histological studies reveal accumulations of fibroblasts or contractile myofibroblasts [25]. Although hypotheses have been proposed, it is still not entirely clear if and how these cells contribute to the development of connective tissue-induced contractures [26]. Recent findings also indicate that the fascial epimysium is pivotal in the pathogenesis of delayed onset muscle soreness (DOMS), which occurs following intense physical activity without adequate preparation [27]. In addition, increased ECM density may contribute to myofascial pain [28,29]. Painful contractures are especially associated with an increase in fascial tissue thickness, resulting in increased relative stiffness [30]. 

It has also been suggested that nonspecific back pain may be mediated, at least in part, by fascial structures [31]; the thoracolumbar fascia, in particular, absorbs a significant portion of the mechanical force transmitted during lumbar flexion and is rich in nociceptive nerve endings [31]. Tears, microlesions, and mechanical irritations of the thoracolumbar fascia can cause malfunctions and painful contractures. The same applies to the musculo-connective structures of the hip, where a significant number of nerve endings have been found not only in the skin (64.0 ± 5.2/cm^2^), where they are most concentrated, but also in the subdermal tissue (24.0 ± 1.4/cm^2^), superficial fascia (33.0 ± 2.5/cm^2^), and deep fascia (19.0 ± 5.0/cm^2^) [32]. It is interesting to note that in men with chronic back pain, there is a tendency toward increased thickness of the thoracolumbar fascia compared with healthy subjects [33]. Furthermore, a reduction in shearing movement, or the ability to glide during passive lumbar flexion, has been documented in this fascial structure in relation to the underlying musculature in both sexes [33]. According to Stecco et al. [29], one of the issues with fascial system gliding, which causes densification and pain, is partly due to an alteration in the production of hyaluronic acid, an important lubricant located between fascial layers.

Myofascial pain syndrome

Although experts in the field have not reached a consensus on the etiology and pathogenesis of myofascial pain syndrome [34], primarily due to the lack of specific laboratory markers and imaging evidence that would enable a unified diagnostic criterion, in general, the term myofascial pain syndrome describes sensory, motor, and autonomic symptoms attributed to myofascial trigger points (MTrPs) [35,36]. MTrPs, which consist of numerous contraction knots, are defined as highly sensitive areas within taut, discrete bands of hardened muscle, leading to localized and referred pain along with other symptoms. Each contraction knot appears as a section of muscle fiber characterized by markedly contracted sarcomeres and an increased fiber diameter. The integrated MTrP hypothesis suggests that, in cases of myofascial pain, motor endplates release excessive acetylcholine, histopathologically indicated by shortened sarcomeres [37]. These zones of intense focal sarcomere contraction have been documented in both animals and humans. Although MTrPs are thought to be quite prevalent, there is limited quality literature available on their exact prevalence [38,39].

MTrPs can be divided into active or latent types. An active MTrP causes a clinical pain disturbance. It is always tender and produces recognizable pain upon compression. It inhibits the full extension of the muscle, weakens the muscle, and mediates a local contraction response of muscle fibers when adequately stimulated [35]. A latent MTrP is clinically asymptomatic and only painful when palpated with some force. A latent MTrP may have all the other physical characteristics of an active MTrP and always presents within a tight band that increases muscle tension and limits the ROM [40].

The underlying mechanisms of MTrPs have only recently started to be elucidated. Jarvholm et al. [41] report that elevated intramuscular pressure in the supraspinatus substantially restricts local muscle blood flow, likely resulting in hypoperfusion and ischemia in the area. The partial oxygen pressure (pO_2_) within regions of muscle hardening, termed myogeloses - presumed clusters of multiple MTrPs - has been found to be extremely low (near zero) at the center of an MTrP region [42]. This notably low pO_2_ is likely associated with a deficiency in adenosine triphosphate (ATP), a factor that may contribute to the localized hypercontractions (contractures) observed in these areas. ATP is essential for breaking myofilament bonds and stopping muscle contraction. Current data on myofascial pain indicate that the discomfort linked to active MTrPs may be indirectly related to reduced blood supply in the affected region. Hypoperfusion, for instance, can lead to low pO_2_ and contractures. Additionally, it is known that low pO_2 _significantly promotes the release of bradykinin, which sensitizes muscle nociceptors.

Since capillary pressure ranges from around 35 mmHg at the beginning (arterial side) to approximately 15 mmHg at the end of the capillary network (venous side), capillary blood flow is transiently obstructed during muscle contractions [35]. Blood flow immediately restores with relaxation, consistent with its normal physiological mechanism. In dynamic rhythmic contractions, intramuscular blood flow is improved by this contraction-relaxation rhythm, also known as the muscle pump. However, during sustained muscle contractions, muscle metabolism is highly dependent on oxygen and glucose levels, which are reduced due to altered vascular dynamics. Contractions performed at 10% to 25% of maximal voluntary contraction are sufficient to produce high intramuscular pressures that significantly compromise intramuscular blood flow [35]. 

According to Bron and Dommerholt [35], these increases in pressure gradient, even during low-level efforts, producing hypoxia, can contribute to the formation of MTrPs and ultimately to the development of pain symptoms [25]. Often referred to in the literature as myofascial pain syndrome [43], its symptomatology involves the muscular, sensory, motor, and autonomic nervous systems, caused by the stimulation of MTrPs [29].

Fascia and the simplified model of force transmission

**Figure 1:**
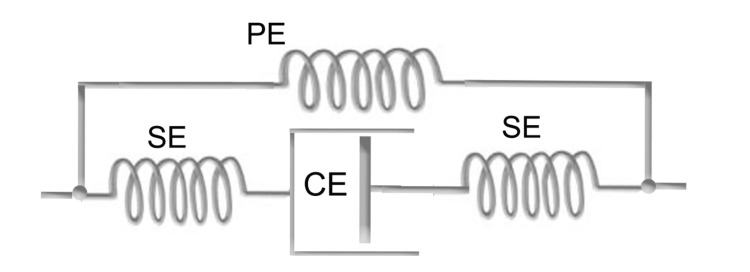
Schematization of myofascial system elements A schematic representation of a finite element model based on Hill's three-element model (1938) illustrates muscle, fascia, and tendon in an anatomically arranged lower limb structure. In this simplified model, the muscle and tendon are aligned in series, while the fascia is arranged in parallel within the leg. The elements are defined as follows: contractile element (CE), series element (SE), and parallel element (PE). The model is adapted from the works of Huijing [26] and Stecco [9]. Image credit: Author Saverio Colonna

In the functional classification of the fascial system [16], as previously outlined, one of the main roles is the transmission of forces. To understand how the fascial system performs the task of force transmission, in terms of both stability and movement, we must refer to Hill's model. One of the most common and simplified versions of the muscle model proposed by Hill [44] consists of three elements: a contractile (active) element in series with an elastic (passive) element and both in parallel with another elastic (passive) element (Figure 1). It is the contractile element that is responsible for the change in muscle length and the generation of force when the muscle is activated. 

Although this simplified interpretation is not unanimously accepted, as it is considered one-dimensional [25], it remains a useful model to explore this topic in greater depth in this article. Its basic structure is schematically composed of (a) contractile component, typically identified with the sliding filaments of actin and myosin, where the force generated depends on the number of active cross-bridges between these filaments; (b) connective component, often linked to the intrinsic elasticity of the myofilaments and cross-bridges, as well as the tendons; and (c) parallel component, associated with the elasticity of connective tissues such as the epimysium, perimysium, and endomysium, along with the sarcolemma [45].

According to Huijing [46], the real situation becomes further complicated because these individual units in turn combine in series and in parallel. While the role of the in-series connective systems is to transfer, usually to the bone, the tension developed by the shortening of the muscle contractile component, the parallel component is responsible for energy recycling. Indeed, many movements in animals, including humans, are cyclic in nature and are associated with a stretch-shortening cycle of the myofascial complex [44].

Many myofascial systems are built to take mechanical and energetic advantage of the stretch-shortening cycle through their parallel elastic elements, influencing the rate of change of the muscle’s contractile elements [44], storing and releasing potential energy [47,48], and increasing work output during the shortening phase through mechanisms of residual force enhancement [49]. The effectiveness of energy storage in fascial tissue is further exemplified by animals such as gazelles and kangaroos, which use the fascial tissue in their lower limbs as an elastic spring [50]. Another example can be observed in athletes with below-knee amputations, who, using a passive system made of elastic metal blades that essentially replace the parallel myofascial component, are able to reach high speeds during running [50,51].

Myofascial pain syndrome - new etiopathogenetic hypothesis

The hypothesis we propose is that myofascial pain syndrome, caused by the formation of MTrPs, may be linked to an altered load of connective systems, as schematized by Hill's model [44]. In the case of incorrect use of parallel connective systems, which are crucial for joint stability, the serial fascial system may be compensating through muscle activation. This activation is constant, not for dynamic purposes (i.e., for producing movement), but for static purposes related to stabilization. Muscle activation within a non-extensible connective sheath may increase internal pressure, reducing blood flow as proposed by Jarvholm et al. [41] in the supraspinatus muscle. This mechanism seems plausible in cases of lower back pain (LBP) where the absence of the flexion relaxation phenomenon (FRP) represents the compensation of serial connective systems, with associated muscle activation observed electromyographically during trunk flexion postures.

FRP, as studied by Floyd and Silver [52] in maximum trunk flexion during upright standing, has also been observed in subjects during slumped sitting postures [53,54]. These postures are commonly adopted during daily life, especially given the time many people spend sitting in chairs, sofas, and armchairs. Such compensatory muscle contraction may trigger a vascular crisis, which may in turn lead to the formation of MTrPs, as identified via ultrasound in the thoracolumbar fascia and erector spinae muscles [55,56]. The results of a study [57] focusing on ultrasound measurements of the thickness of the thoracolumbar fascia and multifidus muscle indicate that individuals with chronic LBP exhibit thickening of the thoracolumbar fascia and thinning of the lumbar multifidus muscle compared with healthy control groups. In particular, an increase in thoracolumbar fascia thickness was correlated with pain intensity, while a reduction in multifidus muscle thickness was associated with decreased lumbar flexion capacity. The authors emphasized the importance of incorporating tailored rehabilitation regimens for LBP patients that target both fascial and muscular components. Another study [58] confirms the reduction in multifidus thickness in patients with LBP.

Fascial treatment - stretching

Many studies suggest treatments aimed at stretching the fascia [59], including manual techniques [60,61] and stretching exercises [3,62]. The goal of these therapies is to modify the mechanical properties of the fascia, such as density, stiffness, and viscosity so that it can more easily adapt to physical stress [63,64]. In fact, manual therapists report a localized release of tissues following the application of slow manual force to abnormally tense fascial areas [64,65]. These outcomes have been explained as a disruption of fascial cross-links, a transition from a gel state to a sol state in the ECM, as well as other passive viscoelastic changes in the fascia [64,65].

Therapeutic fascial stretching

In the early 1900s, Sherrington [66] defined the underlying concepts of neuromuscular facilitation and inhibition. These later led to the development of clinical stretching techniques described by Kabat [67,68] as proprioceptive neuromuscular facilitation (PNF). PNF is based on neurophysiological phenomena, particularly reciprocal inhibition: the process wherein, when a muscle group is activated, its antagonist is inhibited. Initially, PNF techniques were used for the rehabilitative treatment of patients with spasticity and paresis, facilitating muscle stretching. Soon after, the therapeutic use of PNF was extended to patients with conditions of non-neurological origin [69,70], including more common complaints treated in sports medicine [71].

Two commonly used PNF-stretching techniques are contract-relax (CR) and contract-relax followed by agonist contraction (CRAC). During the CR technique, the therapist passively brings the target muscle group of the patient - the group to be stretched - to the point of maximal resistance, referred to as the barrier [72], where further stretching or ROM is limited. In this position, the treated muscles are contracted isometrically for a few seconds and then stretched to a new barrier.

Some authors further differentiate between CR techniques and the hold-relax (HR) method, depending on the type of contraction used before stretching [73-75]. Theoretically, CR involves an isotonic contraction resisted by the therapist, while HR requires a resisted isometric contraction [76,77]. Both methods, rooted in the PNF approach, are applied with the intention of stimulating sensory receptors that provide information about body position and movement to facilitate the desired movement [77]. In both cases, the joint or body part is actively or passively repositioned to the new limit of the ROM following contraction [77]. The primary mechanism underlying this technique is postulated to be the inverse myotatic reflex, also known as autogenic inhibition, due to the action of Golgi tendon organs (GTOs) [78]. In autogenic inhibition, maximal muscle contraction activates the GTOs - structures sensitive to force - thereby inhibiting the alpha motor neurons of the same muscle via type-Ib inhibitory interneurons.

A 2006 review [73] reports that these techniques often vary in their descriptions and are referred to by different names. For example, the CRAC technique consists of two phases: the first is identical to the CR technique, while the second phase adds a contraction of the antagonist muscle group as the therapist stretches the target muscle group. The CRAC technique appears to exploit the myotatic reflex, meaning that the increased discharge frequency of the spindles in the antagonist muscle (due to isometric contraction) stimulates Ia inhibitory interneurons, which in turn inhibit the alpha motor neurons of the antagonist muscles [79]. This should lead to a relaxation of the activated muscles and/or a reduction in the amplitude of the muscle stretch reflex response [80]. Kabat [68] attributed this to the induction resulting from the second phase and used it to develop a PNF strengthening technique, which became known as "antagonist reversal" [68,81]. Several studies [73,82-84] have reported that PNF stretching techniques led to greater increases in ROM compared with static or ballistic stretching. This response seems to be due to the relaxation of the muscle being stretched, as a result of reflex inhibition [85,86].

The hypothesis that PNF exerts its effects through muscle activity inhibition has been challenged by several researchers. Using surface electromyography (EMG), Moore and Hutton [86] studied the relative level of relaxation of the hamstring muscles achieved during different types of stretching. They found that the CRAC technique not only produced the greatest increase in ROM but also significantly higher EMG activity compared with the static or CR technique. Subsequent surface EMG studies [87-89] evaluating various stretching techniques confirmed, as defined by Etnyre and Abraham [90], the apparent paradox that, when using PNF stretching techniques, the greatest ROM gains coincide with increased EMG activation of the stretched muscle, rather than a reduction.

Furthermore, several years ago, Chalmers [91] questioned the neurophysiological foundations of CR, particularly the role of GTOs. After this article appeared, several other authors [92-94] also expressed doubts about the actual mechanisms underlying PNF techniques and stretching methods in general. Traditionally, it has been considered that GTOs serve as "safety devices" that help prevent excessive force during muscle contraction [95]. When the forces of muscle contraction and external factors combine to a point where injury to the muscle, tendon, or bone becomes possible, GTOs generate inhibitory postsynaptic potentials on the cell bodies of the agonist motor units [96-98]. According to Moore [93], recent research has demonstrated that these concepts regarding GTO physiology are inaccurate. The new evidence can be summarized as follows: (a) GTOs respond throughout the entire ROM, even to weak active and passive contractions, although they are much less sensitive to passive contractions than active ones. (b) GTO impulses likely reach the cerebral cortex, informing the spatial position of the limbs. (c) GTOs and their autogenic inhibition reflex reduce, but do not deactivate, the excitability of the motor neurons and the innervated muscle. (d) During CR stretching, the GTO autogenic reflex induces a momentary inhibition that persists only for the duration of the active muscle contraction. Therefore, it is unlikely that GTO activity significantly influences the subsequent relaxation phase, as proposed in earlier literature [96-98].

The cause of the hypothesized change in stretch perception with PNF stretching remains unknown. Increased stretch tolerance has been proposed to explain the acute increases in ROM observed with static stretching and as the basis for the greater ROM in individuals with greater relative physiological flexibility, particularly in the hamstring muscles [98,99]. However, if we simply attribute the increased ROM induced by stretching to greater pain tolerance during the stretch, this cannot explain how, in addition to increased joint mobility, there is also a reduction in "muscular stiffness" immediately after exercise, as assessed by ultrasound shear-wave [100]. Other studies using the same method have confirmed the acute reduction in muscle stiffness at rest following stretching [101-103].

Considering all this data, the question put forward by Carla Stecco in her article [3] becomes more plausible: "Fascial or muscle stretching?"

Neural aspect of stretching

The global relationship between the nervous system and the fascial system is highly complex. Starting with the simple constitution of what is referred to as nervous tissue, it actually involves a significant participation of supporting fascial tissue (endoneurium, perineurium, and epineurium). Further complicating the picture is the response of these fascial structures to mechanical stress in relation to the activity of the autonomic nervous system (sympathetic and parasympathetic systems), which certainly plays a role in the determination of baseline tension states. Based on the current information available, there is insufficient data to fully define these relationships. For this reason, we will limit our discussion to the hypothesized afferent responses of fascial system receptors, specifically regarding force transmission. 

According to Schleip [104], the immediate fascial plasticity observed after manual treatment cannot be explained solely by the mechanical properties of the connective tissue system. The fascia is densely innervated by mechanoreceptors, which include Ruffini endings and a rich network of interstitial receptors with a high reactivity to tangential pressure. The manual stimulation of these sensory endings by the therapist’s manipulation likely leads to changes in the tone of motor units mechanically linked to the tissue involved. Therefore, it is not just mechanical action that is seen but also profound changes in the autonomic nervous system triggered by the stimulation of these receptors.

Supporting Schleip's proposal [104] is the work of Guissard and Duchateau [105], whose title “Neural aspects of muscle stretching” clearly reflects the authors’ stance. The idea that stretching interacts not only with the muscle component but also with the nervous component is not a recent discovery. To our knowledge, the earliest data date back to the work of Robinson et al. in 1982 [106], when the authors used the Hoffman neurological reflex [107] evaluated with EMG to investigate the effect of stretching, which was erroneously termed muscular, referring to muscle as the tension-producing system. 

Unlike rapid muscle stretching, static stretching does not enhance reflex activity in the stretched muscle but instead reduces spinal reflex excitability. This inhibitory effect can be evaluated in the soleus muscle by measuring the Hoffmann reflex (H-reflex) and the tendon reflex (T-reflex) through EMG. The H-reflex is triggered by electrically stimulating the Ia fibers of the tibial nerve in the popliteal fossa [108], whereas the T-reflex is elicited by tapping the Achilles tendon with a reflex hammer.

Myofascial stretching and Hill's model

**Figure 2:**
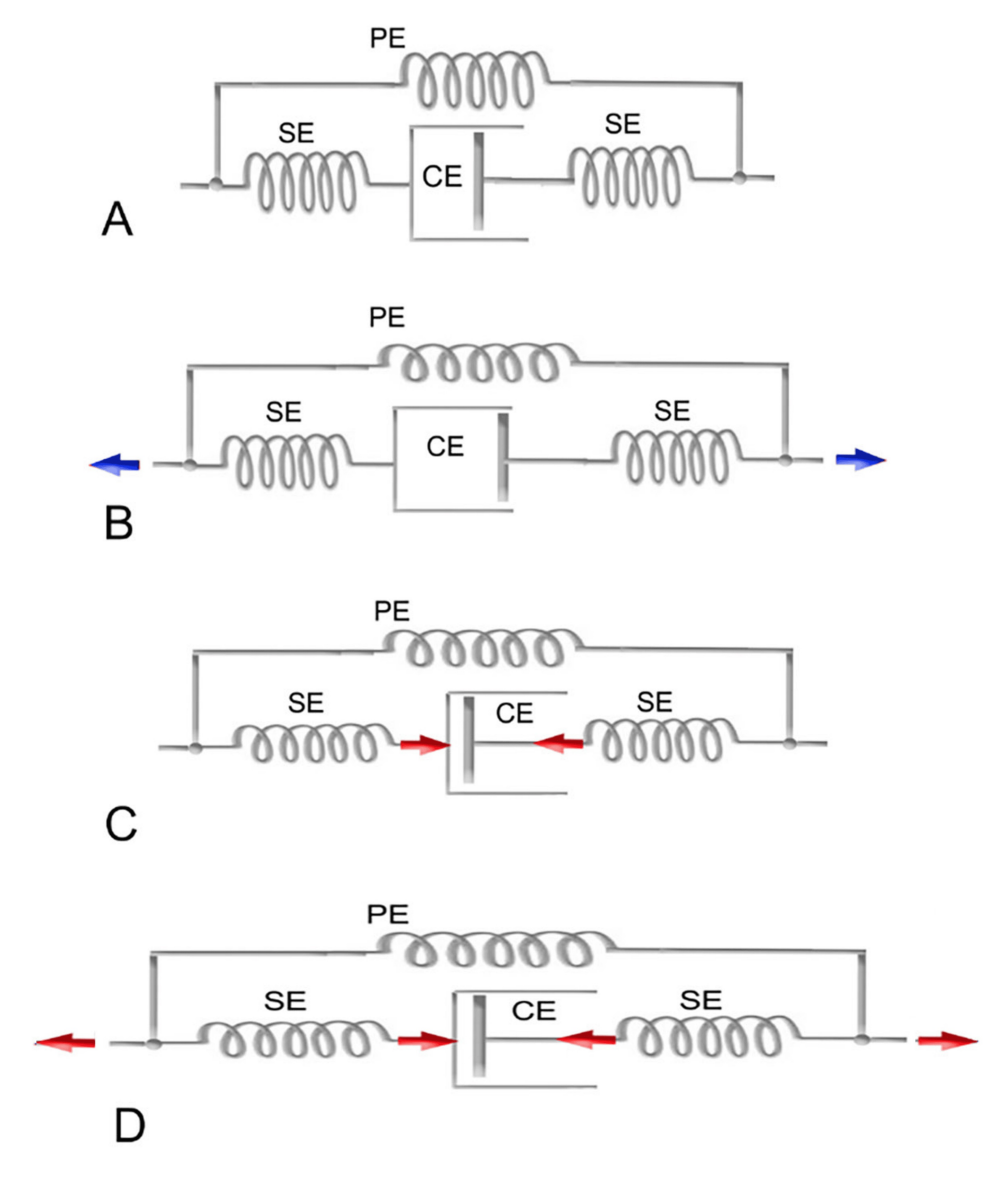
Decomposition of tensile loads across different fascial components (A) Relaxed position: The myofibers are in a relaxed state, with the muscle at its resting length. None of the fascial elements are experiencing stretch or tension. (B) Classic passive stretching: The fascial tissues in parallel with the muscle fibers are stretched; however, the fascial tissues in series with the muscle fibers remain underloaded, as most of the force in this series pathway is absorbed by the relaxed muscle fibers. (C) Typical muscle activity: The muscle fibers are contracted, with the muscle at its stretched length, which applies tension to the fascial tissues arranged in series with the muscle fibers. (D) Combined model of the two previous states (B and C) in a sequential manner: The first part involves the contraction of muscle fibers of the targeted myofascial for approximately 6-15 seconds, which tends to elongate the connective component in series. During the post-activation relaxation of the targeted myofascial system, the antagonist is activated for about 6-15 seconds, increasing the distance between the muscle's insertion points and thereby also stretching the parallel fascial component. The cycle is repeated 3-5 times. In the condition represented in (C), the isometric contraction of antagonist muscles is superimposed, further increasing the distance between the muscle insertion points and thereby placing greater tension on the parallel elastic component. The position is maintained for 6-15 seconds. CE: Contractile element; SE: Series element; PE: Parallel element. Image credit: Author Saverio Colonna

According to Shleip and Müller [109], different stretching styles seem to target the various components of fascial tissue, as described in Hill's model. Figure 2 illustrates some of these different target tissues affected by various loading regimes.

Traditional weight training loads the muscle within its typical ROM, thereby reinforcing the fascial tissues aligned in series with the active muscle fibers. Additionally, the transverse fibers within the muscle sheath are also stretched and activated. However, minimal effects can be expected on extramuscular fascia and on those intramuscular fascial fibers arranged parallel to the active muscle components, as proposed by Huijing [46]. On the other hand, classical stretching, in which the muscular contractile component is inactive, will show minimal effects on those fascial tissues arranged in series with the active muscle component. The reason is that relaxed myofibers, being much more elastic than their fascial/tendinous extensions arranged in series, can absorb most of the stretch [110]. However, slow, light, and constant stretching provides good stimulation for tissues like the extramuscular fascia and the intramuscular fascia arranged parallel to the myofibers.

In light of this presentation, a question arises: Is it possible that different stretching methods act on different components of fascial connective tissue?

**Figure 3:**
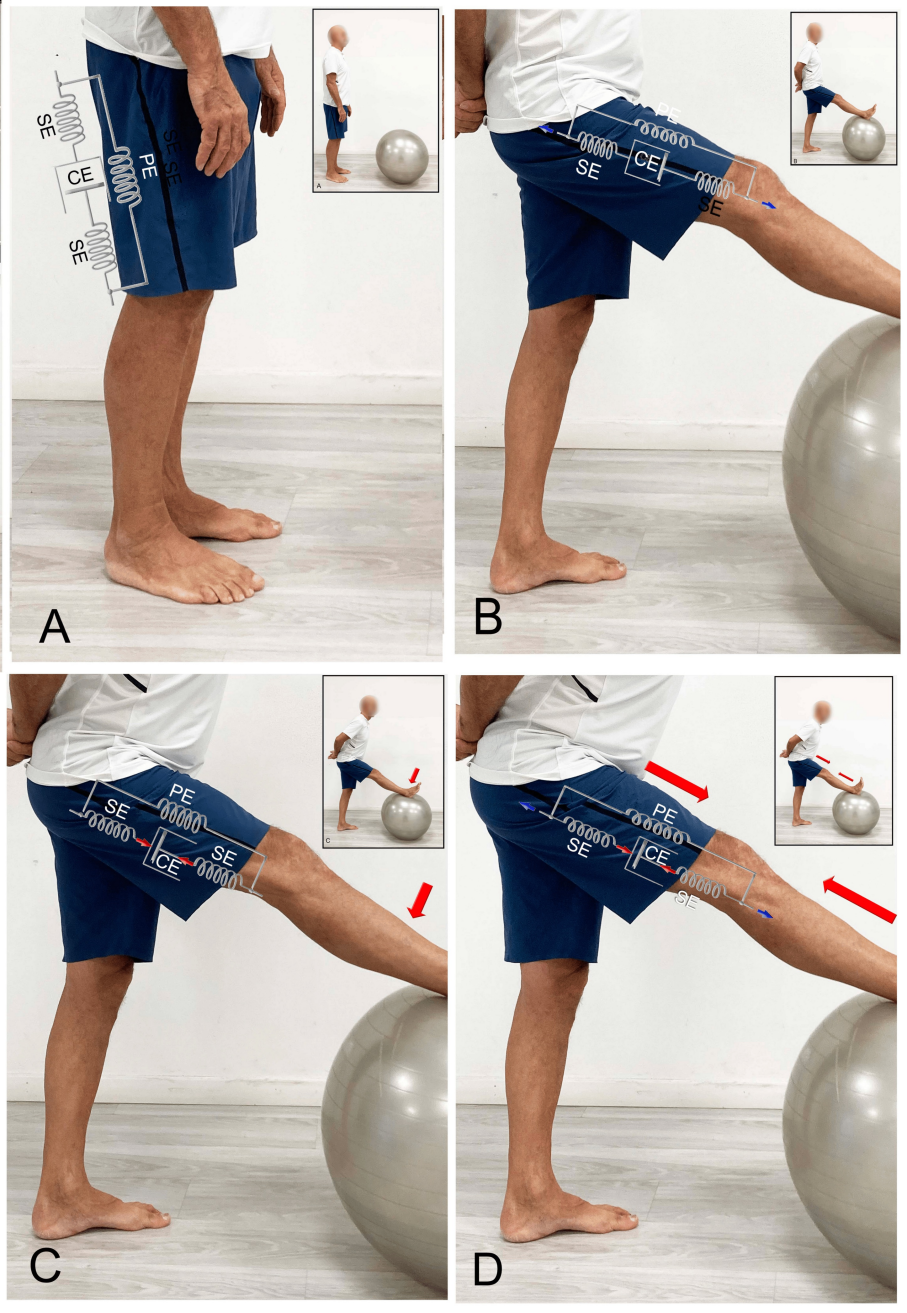
Examples of hamstring stretching (A) Subject in neutral tension condition, (B) passive hamstring stretching with classic method, (C) CR method, and (D) CRAC method. CR: Contract-relax; CRAC: Contract-relax followed by agonist contraction; CE: Contractile element; SE: Series element; PE: Parallel element. Image credit: Author Saverio Colonna

The classic method proposed in the 1980s by Anderson [111] involves passively stretching the myofascial structures by separating the insertion points (Figure 3B). In this situation, the first barrier to stretching is the parallel fascial component. Indeed, the muscular contractile component, due to the greater elasticity of the relaxed myofibrils, reduces the tension induced by the stretch on the series fascial component. Therefore, this method might stimulate the elongation only of the fascia arranged in parallel.

The CR stretch, by activating the contractile component of the muscular system while in the stretched position produced by the previous method, may tend to primarily load tension onto the series fascial component in the Hill model, while simultaneously reducing the tension on the parallel system (Figure 3C). On the other hand, the CRAC technique, especially in the second phase (Figure 3D), which involves the contraction of the antagonist anterior muscles, further separates the insertion points of the posterior fascial system, involving the parallel fascial component.

This model is novel because, instead of relying on the neurophysiological principles outlined by Sherrington (particularly considering the reconsideration of the function of the Golgi tendon organ previously discussed), it is primarily based on the effect of mechanical load on fascial components arranged in series and parallel. This model should also be considered when applying certain manual therapy techniques, such as muscle energy technique (MET) [112], which are directed toward the fascial system.

## Conclusions

This article is the first, to our knowledge, to address fascia dysfunction in two modalities: excessive stiffness and reduced stiffness. This first part of an investigation of physical exercise and the fascial system provides an introduction to the composition of fascia and an overview of the methods proposed in the literature to address dysfunctions caused by excessive fascial rigidity. In addition, the neurophysiological principles underlying traditional muscle stretching are questioned, exploring a model that shifts the focus more toward the fascial component rather than the muscle's contractile component. By revisiting models that schematize the fascial system, such as Hill's finite element model, it attempts to apply these to the techniques most commonly proposed in the literature. This article presents a new hypothesis on how MTrPs may develop as a result of imbalanced tension loading on the fascial components arranged in series and parallel to the contractile muscular component. If future research were to confirm these hypotheses, it would follow that, for an effective and complete treatment of fascial system stretching, all three methods should be used in sequence. This model should also be considered when applying certain manual therapy techniques.
